# The Neutrophil-to-Lymphocyte Ratio in Patients with Spinal Cord Injury: A Narrative Review Study

**DOI:** 10.3390/medicina60101567

**Published:** 2024-09-25

**Authors:** Seyed Ahmad Naseri Alavi, Mohammad Amin Habibi, Seyed Hamed Naseri Alavi, Mahsa Zamani, Andrew J. Kobets

**Affiliations:** 1 Department of Neurological Surgery, School of Medicine, Emory University, Atlanta, GA 30322, USA; 2 Department of Neurosurgery, Shariati Hospital, Tehran University of Sciences, Tehran 1441987566, Iran; mohammad.habibi1392@yahoo.com; 3 Faculty of Medicine, Guilan University of Medical Sciences, Rasht 4144666949, Iran; hamednaserialavi@gmail.com (S.H.N.A.); mahsazam@gmail.com (M.Z.); 4 Department of Neurological Surgery, Montefiore Medical, Bronx, NY 10467, USA; akobets@montefiore.org

**Keywords:** trauma, spine, spinal cord injury, SCI, N/L ratio, neutrophil-to-lymphocyte ratio

## Abstract

*Background and Objectives***:** Traumatic spinal cord injury (SCI) is a devastating condition that occurs in two phases: primary and secondary injury. These phases contribute to changes in blood vessels and the influx of inflammatory cells such as neutrophils and lymphocytes. The biomarker known as the neutrophil-to-lymphocyte ratio (NLR) has been suggested as being highly valuable in predicting outcomes for patients with traumatic brain injury, acute ischemic stroke, and traumatic spinal cord injury. Therefore, this review study aims to investigate the prognostic value of the NLR in predicting outcomes for patients with SCI. *Materials and Methods*: A thorough review of relevant articles was conducted using Mesh keywords in Medline via Embase, PubMed, Google Scholar, and Scopus from 2000 to 2023. The search was conducted using the Preferred Reporting Items for Systematic Reviews and Meta-Analyses (PRISMA) checklist. After reviewing the articles and applying inclusion and exclusion criteria, only relevant articles were included in the study. *Results*: In the initial search, 41 papers were identified. After applying exclusion criteria, only three clinical studies remained for review. It is still debatable whether the NLR can serve as a cost-effective, readily available, and independent predictive factor for both mortality and recovery outcomes in patients with traumatic spinal cord injuries. *Conclusions*: Our study demonstrates that NLR, a readily available and inexpensive marker, can serve as an independent predictor of both mortality and recovery outcomes in patients with traumatic spinal cord injury. To reach a conclusive decision, additional data are required.

## 1. Introduction

Traumatic spinal cord injury (SCI) is a debilitating disease with devastating consequences to a patient’s life due to its significant functional and economic burden. Approximately 11,000 new cases are estimated to occur from SCI each year in the US [1]. Fortunately, recent advancements in managing patients suffering from SCI have improved their survival rate; however, they still have a poor outcome compared to the general population [2].

Injury following trauma to the spinal cord has two stages: primary and secondary injury. Primary injury occurs due to the initial traumatic event leading to structural damage to the vertebral column, including dislocation and the fracture of vertebrae with subsequent spinal cord compression or transaction [3,4]. The secondary injury is defined as a series of changes that begins within a few minutes of the initial injury, causing continuous and progressive damage to the spinal cord, leading to neurological dysfunction. Further, it is composed of vascular changes, including ischemia derived by the destruction of the microvascular supply of the spinal cord; severe hemorrhages of blood vessels with the resultant influx of inflammatory cells, cytokines, and vasoactive peptides [5,6]; inflammatory response, with increases in the level of proinflammatory cytokines leading to the infiltration of inflammatory cells such as neutrophils, lymphocytes, and macrophages into the spinal cord, that remain in the cord for weeks [1]; chemical change and ionic derangement, including calcium influx into cells; and the release of potassium into the extracellular fluid as a result of the necrosis of neurons and glia due to ischemia cellular dysfunction, including necrotic cell death, lipid peroxidation, and free radical formation [2].

The secondary injury cascade may last months past the initial injury and its damage is often more than that caused by the initial damage [3,4]. Inflammation, one of the main contributors to secondary injury, is generally considered detrimental to the spinal cord after injury. However, recent studies show that inflammatory cells such as neutrophils play important roles in SCI recovery [4].

The neutrophil-to-lymphocyte ratio is a biomarker that has been proposed to have great value in predicting the outcome of many neurological disorders, including traumatic brain injury, acute ischemic stroke, and traumatic spinal cord injury [7,8,9]. Moreover, it has been shown that higher circulating neutrophil counts are associated with more severe injuries and worsened neurological outcomes in patients with SCI [6]. Another study showed that there is a positive correlation between the extent of neutrophilia in patients suffering from SCI and New Injury Severity Scores (NISS). Moreover, the extent of neutrophilia has an inverse correlation with the neurological outcome (American Spinal Injury Association Impairment Scale) [10]. Hence, this review study aimed to investigate the role of the NLR in SCI to investigate the value of the NLR in the prediction of the outcome of patients with SCI.

### Importance and Novelty

Recent studies have shown that the neutrophil-to-lymphocyte ratio (NLR) can be a reliable factor in patients with spinal cord injury (SCI). The objective of this study is to introduce and emphasize a cost-effective and easily accessible lab test for neurosurgeons, especially in lower-income and developing countries. It is worth noting that in many developing countries, with a high mortality rate following road accidents and limited access to intensive care and trauma centers, such simple and affordable ratios can prove to be extremely useful.

## 2. Materials and Methods

### 2.1. Object

The systematic review was conducted by the suggested procedures outlined by the Preferred Reporting Items for Systematic Reviews and Meta-Analyses for Protocols (PRISMA).

### 2.2. Databases and Search Strategy

**Figure 1:**
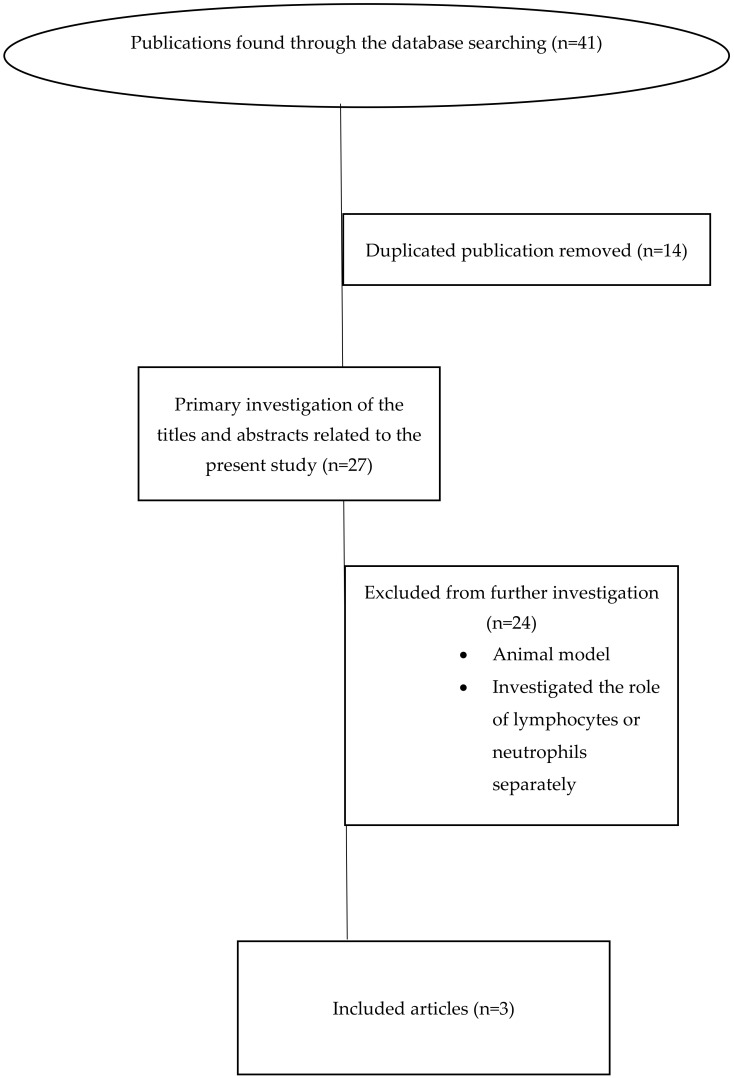
Search strategy to find clinical studies investigating role of N/L ratio in patients with SCI.

To the best of our knowledge, we identified all the studies on the effects of the neutrophil-to-lymphocyte ratio in patients with spinal cord injury by a systematic search of the literature and a hand search of different electronic databases and the literature, including PubMed, Scopus, Web of Science databases, Google Scholar, and EMBASE for studies which had been published from 2000 to 2023. The specific medical subject headings (MeSHs) in the search were as follows: “Spinal Cord Injury” OR “Traumatic Spinal Cord Injury” OR “TSCI” OR “Spine Injury” OR “SCI”, AND “N/L Ratio” OR “Neutrophil to Lymphocyte Ratio” OR “Neutrophil-to-Lymphocyte Ratio”. All in vivo and in vitro studies including case reports, letters to editors, review articles, and other relevant data were enrolled in the study. The titles and abstracts of all articles attained from the databases were reviewed by two senior independent authors to investigate eligibility based on the aim of the study. Then, full texts of the related articles were enrolled; duplicated and non-related articles were excluded. Finally, three clinical studies were enrolled and reviewed (Figure 1).

### 2.3. Inclusion and Exclusion Criteria

The inclusion criteria in the present study were as follows: all clinical studies in English from January 2000 to October 2023 that investigated the role of the N/L ratio following SCI were included. The two reviewers searched the databases mentioned before based on the title and abstract of the studies. On the other hand, the papers on animal models and articles that investigated the role of neutrophils and lymphocytes separately in patients with SCI were excluded from the study.

### 2.4. Study Outcomes

The primary outcome of this review was evidence of the important role of the NLR in patients following SCI.

### 2.5. Spinal Cord Injury

Spinal cord injury (SCI), a life-threatening medical condition, is one of the most significant kinds of traumatic damage, with substantial socioeconomic burdens on the patients, caretakers, and governments. It is characterized by a chronic, inflammatory condition at the lesion site, with poor outcomes whenever a neurological defect occurs [11]. Like traumatic brain injury, SCI is induced by traumatic injuries and is responsible for the primary phase. Subsequently, inflammation begins, and the secondary injury persists, which shows the well-known and primary role of inflammatory responses in the further stages of injury [12].

### 2.6. The Neutrophils’ Role after SCI

The neutrophil is a subtype of white blood cell (WBC) and plays a role in CNS dysfunction and neuroinflammation by producing extracellular proinflammatory mediators [12]. The first study that demonstrated the influence of neutrophils in inflammatory CNS disorders took place back in 1998 and was on the experimental autoimmune encephalomyelitis model [13]. Afterward, further studies focused on the role of neutrophils in another clinical condition and established the neutrophil-to-lymphocyte ratio (NLR) for predicting clinical outcomes. The NLR demonstrates the level of balance between neutrophils and lymphocytes, representing systemic inflammation [14], and has been recognized as a clinical indicator in several inflammation-based diseases such as autoimmune diseases [15], neurodegenerative disorders [16], cancer [17], stroke [18], and cardiovascular disorders [19].

It has been observed that young patients with SCI have a good prognosis when neurological defects are absent [11]. Accordingly, predicting the prognostic outcome would be a crucial factor for the long-term treatment of patients with SCI and decreasing socioeconomic burdens for them. Up to now, several prognostic indicators at admission have been used for the prediction of outcomes of SCI patients, like the American Spinal Injury Association Impairment Scale (AIS), Glasgow Coma Scale (GCS), Charleson Co-morbidity Index (CCI), platelet counts, and the international normalized ratio (INR) [20]. Furthermore, some models of the available indicators have been made to provide a higher potential predictive system that is even ineffective in the prognostication of SCI patients [21]. However, there have been limitations in this regard, and offering a prognostic indicator with more accuracy, availability, and low cost is justified.

It has been demonstrated that the acute inflammatory response is vast in SCI compared to traumatic brain injury; the number of neutrophils and microglia was also fewer [22]. Following SCI, it is called primary injury, and direct mechanical damage to neurons induces cell death and necrosis [23]. The immune system is activated as a response to the early-stage phase, producing cytokines and chemokines to infiltrate leukocytes at the site of action [24,25]. The secondary injury begins after the initial injury by spreading tissue damage surrounding the initial core site [6]. Numerous mechanisms have been classified, including vascular events, the breaking down of the spinal cord–blood barrier, edema formation, hypoxia and ischemia, perfusion alteration, biochemical events, damage to mitochondria, protease release and energy discharge, cellular events, resident microglia and astrocyte activation, oligodendrocyte apoptosis, and the invasion of macrophages, neutrophils, and lymphocytes [26].

Neutrophils are the first cells presented at the site of inflammation following injury. They are increased within 3 h and remain up to 3 days at the site of infection following SCI [27,28]. Neutrophils are limited to the acute phase of injury, and will vanish gradually in the sub-acute phase [29]; however, their role is controversial [30]. It is shown that neutrophils participate in tissue debris clearance, phagocytosis, the release of inflammatory cytokines, protease and free radicals, the activation of astrocytes, and neuroinflammation initiation [30]. Neutrophils appear to have positive [31] and detrimental effects simultaneously [30] after SCI. Sterling et al. demonstrated that a decrease in the neutrophil count could prevent functional recovery in animals in the acute phase following SCI [31]. They release IL-1 receptor antagonists that protect neurons following injury [32].

### 2.7. The Lymphocytes’ Role after SCI

**Figure 2:**
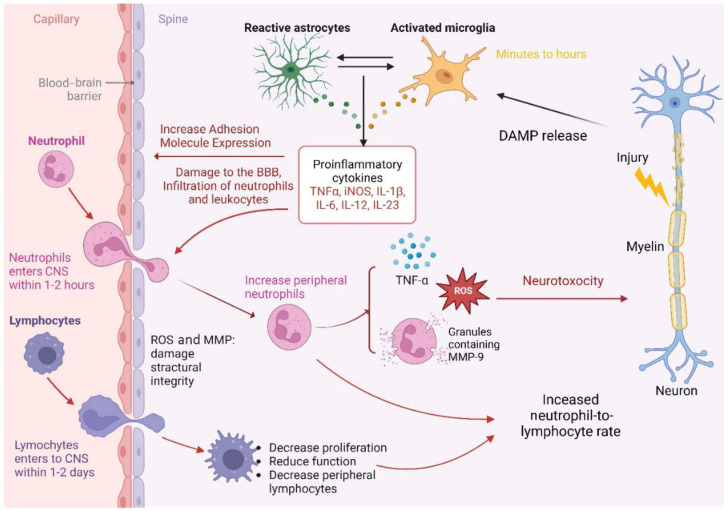
The figure shows the role of neutrophils, lymphocytes, and proinflammatory cytokines following spinal cord injury.

Lymphocytes have been observed to increase in the area of injury within the first week after injury and remain chronic [26,33]. SCI evokes T lymphocytes to the site of injury acutely, and the infiltration affects the exacerbation of survival and neural cell function by producing cytokines and chemokines, including IL-1β, TNF-α, IL-12, CCL2, CCL5, and CXCL10 (Figure 2) [33,34]. It is shown that the elimination of or reduction in the number of T cells by pharmacological intervention, including with Tacrolimus and Cyclosporine, can improve the outcome and functional recovery of damaged tissue following SCI [33]. However, Hauben et al. concluded that T lymphocytes have a crucial role in neuronal tissue following SCI [35]. Conversely, SCI suppresses antibody production in B lymphocytes [36]. The mechanism of B lymphocyte response in SCI is not clear; however, it has been found that the suppression of B cells occurs because of an increase in corticosterone and norepinephrine serum levels [33,36]. Ren et al. showed that B lymphocytes have positive effects on neuron tissue recovery [37]. Of note, the number of lymphocytes is low in the damaged tissue following surgery [22].

### 2.8. The Role of the Neutrophil-to-Lymphocyte Ratio in SCI

NLR is an easy-accessible lab finding calculated as the ratio of the neutrophil to lymphocyte count in the circulating blood. Although the NLR has rarely been evaluated as a prognostic factor of SCI, our review suggests that the NLR could be an independent predictor of mortality in patients with traumatic spinal cord injury. Neutrophils release oxidative and proteolytic enzymes to sterilize the damaged area, preparing it for subsequent ‘repair’, but excessive numbers of neutrophils can cause ‘bystander’ tissue damage. Several studies in animal models of SCI have shown that the blockade of neutrophil influx which limits secondary damage after SCI is ‘neuroprotective’ [27,28,29,30].

A study by Zhao et al. demonstrated the importance of the NLR as a predictor factor in patients with cervical SCI. They investigated 377 patients for a 6-month follow-up period and showed that a higher NLR at admission had a significant relationship with lower AIS grades and poor outcomes [9]. Retrospectively analyzed data from 161 SCI patients admitted to Brisbane’s Princess Alexandra Hospital showed that the degree of intense neutrophilia in SCI patients is positively correlated with New Injury Severity Scores but inversely with the neurological sequel. The multivariate analysis illustrated that acute SCI–induced neutrophilia is an independent predictor of AIS grade conversion failure, with an odds ratio (OR) of 4.16 and an ROC area under the curve (AUC) of 0.82, (*p* < 0.0001). SCI-induced lymphopenia was separately identified as an independent predictor of better recovery (OR = 24.15; ROC AUC = 0.85, *p* < 0.0001). Hence, the results demonstrate the value of the early presentation of neutrophils and lymphocytes to patients’ prognosis for longer-term recovery after SCI [5].

A retrospective cohort study from January 2013 to January 2014 involving 1356 patients admitted to the surgical ICU of a level 1 trauma center showed that the use of the receiver operating characteristic and Kaplan–Meier curves analyzed and revealed that an NLR greater than or equal to these cut-offs could increase hospital mortality, especially in days 2 and 5. So, as a result, the NLR is associated with mortality in critically ill trauma patients [7].

A case–control study illustrates that patients with isolated cervical SCI have significantly higher leukocytosis rates and lymphopenia than controls during the first week post-trauma, and the degree of lymphopenia was significantly associated with the severity of SCI [28,38,39,40,41,42,43].

Some recent retrospective studies carefully examining the WBC response to SCI showed a strong relationship between significantly elevated neutrophil numbers in the blood of patients with an acute traumatic SCI. In contrast to neutrophils, circulating lymphocytes did not increase in number in response to traumatic SCI [5,44,45]. Jogia et al. found that an NLR at 3 dpi has a significant relationship with ICU admission [5].

Considering the prognostic significance of NLR in other immune-mediated disorders and the increasing trend in the prevalence of SCI worldwide [41], it is expected that the NLR can also be applied with prognostic value in outcome predictions of patients with SCI [42]. Here, in this review, we have tried to summarize the clinical aspects of the NLR in the prediction of SCI and highlight the predictive value of the NLR to provide a realistic guide for the management of patients and choose a more appropriate treatment strategy (Table 1). In lower-income, undeveloped, and developing countries, such as Iran, which has a high mortality rate following road accidents, and due to the limited number of ICU-care and trauma referral centers, these simple-, cheap-, and accessible-to-use ratios will show their usefulness [46,47,48]. Additionally, the most recent studies by Zhou et al. and Naseri Alavi have demonstrated that the NLR could be an important predicting factor in patients following SCI [49,50]. In the study by Zhou et al. on 526 subjects with SCI, the sensitivity and specificity of using the NLR were calculated, where the *p*-value = 0.79, = 0.57, respectively [49]. Although our recent study showed the importance of using the NLR in patients following SCI, there was no significant relationship between neutrophil counts at admission and outcome following SCI; however, counts decreased significantly over time [50]. This could be because of the younger average age of our study compared to previous similar studies, with this affecting the NLR [9,49,50,51]. Hence, it remains unclear whether to use the NLR alone or concurrently with other clinical manifestations at admission to predict a reliable outcome in patients with SCI.

### 2.9. Pathophysiology of NLR Role Following SCI

Following SCI, neuroinflammation starts by attracting neutrophils to the injury site, initiating the recovery process through the secretion of chemokines, cytokines, and the phagocytosis of acute cellular debris accumulation. However, neutrophils are a part of the creation of scars and the inhibition of axonal recovery following injury [29,52,53]. Similar to traumatic brain injury, the number of neutrophils increases strikingly while lymphocyte counts remain relatively unchanged in the early stage following SCI. This increase in the NLR possibly leads to cellular injury, scar formation, and damage spinal cord cells following injury [9,49,50].

## 3. Limitations

Due to the limited number of studies investigating the NLR’s role in patients with SCI, further studies are recommended. The limited number of articles investigating the role of the N/L ratio in patients with SCI affects the conclusion of the study; however, we believe that the N/L ratio, as a simple and accessible lab finding, could help neurosurgeons to evaluate patients following SCI in early admission.

## 4. Conclusions

This study’s findings conclusively demonstrate the NLR as an independent predictor of mortality and recovery outcomes in patients with traumatic spinal cord injury. It is a straightforward and clinically accurate measure to calculate, but further investigations are recommended.

## Figures and Tables

**Table 1:** A summary of the studies included in the review, indicating the role of the NLR in predicting functional outcome and mortality in patients with varying degrees of spinal cord injury.

Reference (Year)	Type of Study	Type of Trauma	Number of Subjects	Mean Age _ SD (y) or Range	Sex (M/F)	NL Ratio	Outcome	Additional Data Based on ROC-AUC
Zhao et al. (2020) [9]	Retrospective	Mild	377 (human)	46.05 ± 17.93	212/156	13.28 ± 11.46	The NLR is an independent predictor of outcomes in the 6-month follow-up period in patients with acute cervical SCI	N/A
Zhou et al. (2023) [49]	Retrospective	Mild, Moderate, Severe	526 (human)	52.2 ± 12.5	439/87	N/A	The NLR showed higher diagnostic performance than the platelet-to-lymphocyte ratio, lymphocyte-to-monocyte ratio, and systemic immune-inflammatory index	Sensitivity = 0.79, specificity = 0.57
Naseri Alavi et al. (2023) [50]	Retrospective	Mild, Moderate, Severe	536 (human)	40.93 ± 12.77 (min = 18, max = 64)	399/137	8.43 ± 8.34 (min = 0.68, max = 70.69)	There was no significant relationship between neutrophil counts at admission and outcome following SCI; however, these counts decreased significantly over time.	N/A

## Data Availability

Not applicable.
